# *CYP11B2 T-344C* Gene Polymorphism and Atrial Fibrillation: A Meta-Analysis of 2,758 Subjects

**DOI:** 10.1371/journal.pone.0050910

**Published:** 2012-11-28

**Authors:** Yan-yan Li, Chuan-wei Zhou, Jian Xu, Yun Qian, Bei Wang

**Affiliations:** Department of Geriatrics, First Affiliated Hospital of Nanjing Medical University, Nanjing, China; The University of Texas Health Science Center, United States of America

## Abstract

**Background:**

*Aldosterone synthase (CYP11B2)* T-344C gene polymorphism was found to be correlated with atrial fibrillation (AF) risk. However, the results of individual studies remain conflicting.

**Objective and methods:**

A meta-analysis including 2,758 subjects from six individual studies was performed to explore the correlation between *CYP11B2* T-344C gene polymorphisms and AF. The pooled odds ratios (ORs) and their corresponding 95% confidence intervals (95% CIs) were evaluated by the fixed– or random–effects model.

**Results:**

A significant relationship between *CYP11B2* T-344C gene polymorphism and AF was found under allelic (OR: 1.26, 95% CI: 1.11–1.42, *P* = 0.0002), recessive (OR: 1.99, 95% CI: 1.26–3.14, *P* = 0.003), dominant (OR: 0.903, 95% CI: 0.820–0.994, *P* = 0.036), homozygous (OR: 1.356, 95% CI: 1.130–1.628, *P* = 0.001), and additive (OR: 1.153, 95% CI: 1.070–1.243, *P* = 1.0×10^−10^) genetic models. No significant association between *CYP11B2* T-344C gene polymorphism and AF was found under the heterozygous genetic model (OR: 1.040, 95% CI: 0.956–1.131, *P* = 0.361).

**Conclusions:**

A significant association was found between *CYP11B2* T-344C gene polymorphism and AF risk. Individuals with the C allele of *CYP11B2* T-344C gene polymorphism have higher risk for AF.

## Introduction

Atrial fibrillation (AF) is the most common and damaging arrhythmia in clinical practice. The prevalence of AF increases with age, from 0.5% of people in their 50s to nearly 10% of the octogenarian population [1]. In China, morbidity related to AF is 0.77% in the adult population [2]. AF causes chronic heart failure, tachycardia-induced cardiomyopathy, and increased thrombosis risk, especially cerebral embolism. AF patients have a higher risk for stroke than non-AF individuals. Approximately 15% of stroke cases are caused by AF. Additionally, AF is the first independent risk factor for ischemic stroke in patients more than 75 years old [3]. The death risk of AF patients is 1.5 to 1.9 times that of non-AF patients [4].

Activation of the rennin-angiotensin-aldosterone system plays a key role in AF formation. Aldosterone is the steroid hormone secreted by the adrenal cortex zona glomerulosa, wherein the classic action pathway is to be combined with the aldosterone receptor in the renal distal convoluted tubule and manifolds. Thus, aldosterone exhibits sodium retention and potassium elimination effects that contribute to increased blood volume, blood pressure, left ventricular pressure, left ventricular volume, left atrial pressure, and left atrial volume. Aside from the indirect effect of aldosterone on the cardiovascular system, aldosterone directly affects the heart. Animal experiments suggested that aldosterone possibly induces cardiac hypertrophy and fibrosis [5].

Recent studies have shown that local cardiac tissue could also synthesize and secrete aldosterone. Aldosterone receptors broadly exist in cardiomyocytes, vascular smooth muscle cells (VSMCs), and fibrocytes. The binding of aldosterone to its receptor promotes the differentiation and proliferation of cardiomyocytes and VSMCs. It could also precipitate fibrocyte proliferation and hypertrophy. Hence, collagen production is increased significantly, leading to cardiac fibrosis, remodeling, and arrthythmia. In 1999, Delcayre et al. found that plasma aldosterone could act on angiotensin II receptor, which strengthens the legacy effect after angiotensin II binds to the receptor. Aldosterone could increase the gene expressions of c-fos and c-jun, thereby increasing collagen generation and promoting cardiac fibrosis through activation of the inositol triphosphate pathway [6]. Hence, aldosterone could cause atrial enlargement and fibrosis, which promote atrial structural and electrical remodeling, as well as induce and maintain AF episodes.

Although the pathology of AF has been transited from “locality drive and multiple wavelet foldback” to “locality drive with fibrillatory conduction” and pulmonary wave theory, the mechanism has not been fully clarified yet. Single-nucleotide polymorphisms (SNPs) are DNA sequence polymorphisms caused by single nucleotide variations in the chromosomes, in which at least one allele frequency in the population is not less than 1%. SNPs are the most common human heritable variations [7].

The *aldosterone synthase* (*CYP11B2*) gene located in 8q22 spans approximately 7 kb and contains nine exons and eight introns. *CYP11B2*, which belongs to the cytochrome P-450 gene superfamily, is the catalytic enzyme of the last biochemical reaction step in vivo to synthesize aldosterone [8]. The 344^th^ base thymine (T) is substituted by cytosine (C) at the promoter region, which is the binding site for steroidogenic transcription factor 1. Mutations in the *CYP11B2* T-344C gene could elevate serum aldosterone level and increase type I and III collagen gene expressions, which promote fibrosis and cause inconsistent conduction [9].

Although several studies investigated the association between *CYP11B2* T-344C gene polymorphism and AF, their results remain inconsistent. In 2011, Sun et al. [10] found that *CYP11B2* T-344C gene polymorphism is not associated with AF but may be associated with hypertensive atrial remodeling in a Chinese population [10]. By contrast, Wang et al. [11] reported that *CYP11B2* T-344C gene polymorphism might play a role in the occurrence of AF in Chinese hypertensive patients. In 2008, Amir et al. [12] found that *CYP11B2* T-344C gene polymorphism is predisposed to clinical AF in Israel.

In the present study, a meta-analysis including 1,054 AF patients and 1,704 controls was performed to determine the relationship between *CYP11B2* T-344C gene polymorphism and AF (Supplement S1).

**Table 1:** Characteristics of the investigated studies of the association of the CYP11B2 T-344C gene polymorphism and atrial fibrillation (AF).

Author	Year	Region	Ethnicity	AF			Control			geno-typing	Studydesign	Matching criteria	sample size(AF/control)
				TT	TC	CC	TT	TC	CC				
Amir O [12]	2008	Israel	Asia	13	25	25	54	152	64	PCR-RFLP	Case-control	Age,sex,ethnicity	63/270
Huang M [17]	2009	China	Asia	44	43	10	266	223	40	PCR-SSOM	Case-control	Sex,ethnicity	97/529
Zhang FG [18]	2009	China	Asia	56	59	5	75	43	2	PCR-RFLP	Case-control	Age,sex,ethnicity	120/120
Hu XJ [19]	2010	China	Asia	24	30	5	48	24	5	PCR-RFLP	Case-control	Age,sex,ethnicity	59/77
Sun X [10]	2011	China	Asia	130	157	23	150	138	22	PCR-RFLP	Case-control	Age,sex,ethnicity	310/310
Wang ZF [11]	2011	China	Asia	150	156	99	136	208	54	PCR-RFLP	Case-control	Age,sex,BMI, ethnicity	405/398

AF: atrial fibrillation; *CYP11B2* : aldosterone synthase; BMI: body mass index.

PCR-RFLP: polymerase chain reaction-restriction fragment length polymorphism;

## Materials and Methods

### Publication Search and Inclusion Criteria

The following keywords were searched in electronic databases such as Embase, PubMed, Web of Science, China Biological Medicine Database, and China National Knowledge Infrastructure: “*aldosterone synthase*,” “CYP11B2,” “T-344C,” “atrial fibrillation,” and “polymorphism.” Other relevant studies were also found in the indexed references of the retrieved literatures. The last research was updated on October 5, 2012, with publication years ranging from 2008 to 2011.

**Figure 1:**
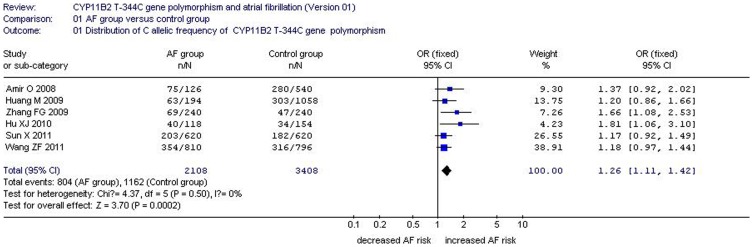
Forest plot of AF associated with CYP11B2 T-344C gene polymorphism under an allelic genetic model (distribution of C allelic frequency of CYP11B2 gene).

**Figure 2:**
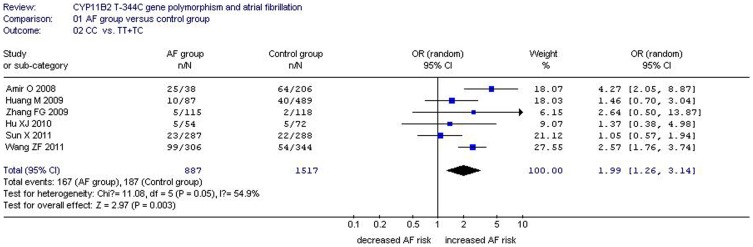
Forest plot of AF associated with CYP11B2 T-344C gene polymorphism under a recessive genetic model (CC vs. TT+TC).

**Table 2:** Summary of meta-analysis of association of CYP11B2 T-344C gene polymorphism and AF.

Genetic model	Group analysis	Pooled OR (95% CI)	P value	Literature number	AF size	control size	*P*_heterogeneity_
Allelic genetic model	whole population	1. 26(1.11–1.42)	0.0002*	6	1054	1704	0.50
Recessive genetic model	whole population	1.99(1.26–3.14)	0.003*	6	1054	1704	0.05*
	Subgroup 1: China	1.71(1.10–2.65)	0.02*	5	991	1434	0.14
	Subgroup 2: Israel	4.27(2.05–8.87)	0.0001*	1	63	270	NA
Dominant genetic model	whole population	0.903(0.820–0.994)	0.036*	6	1054	1704	0.079*
	Subgroup 1: China	0.897(0.814–0.989)	0.029*	5	991	1434	0.049*
	Subgroup 2: Israel	1.032(0.601–1.770)	0.910	1	63	270	NA
Homo genetic model	whole population	1.356(1.130–1.628)	0.001*	6	1054	1704	0.813
Hetero genetic model	whole population	1.040(0.956–1.131)	0.361	6	1054	1704	0.001*
	Subgroup 1: China	1.055(0.965–1.153)	0.238	5	991	1434	0.001*
	Subgroup 2: Israel	0.892(0.699–1.137)	0.355	1	63	270	NA
Additive genetic model	whole population	1.153(1.070–1.243)	1.0×10^−10^ *	6	1054	1704	0.416

* P<0.05; ^*^ ***P*** **_heterogeneity_**<0.10.

Abbreviations:

CI: confidence interval; OR: odds ratio; AF size: the total number of AF cases; control size: the total number of control group; homo genetic model: homozygote genetic model; hetero genetic model: heterozygote genetic model; NA: not applicable.

The studies were selected based on the following inclusion criteria: studies that evaluate *CYP11B2* T-344C gene polymorphism and AF, studies that diagnose AF as episodes ≥ two occasions (>6 months apart) by serial 12-lead electrocardiography (ECG) or 24 h Holter monitoring, case-control or cohort studies published in official journals, and studies that conform to the Hardy-Weinberg equilibrium (HWE). The diagnosis points in ECG were as follows: p wave disappearance and substitution of irregular baseline fluctuation (f wave) with the frequency of 350 to 600 per minute, extremely irregular ventricle rate, and inconsistent QRS complex morphology. The QRS complex would be broader and transformed when the aberrant ventricular conduction appeared. The RR internal is always irregular.

**Figure 3:**
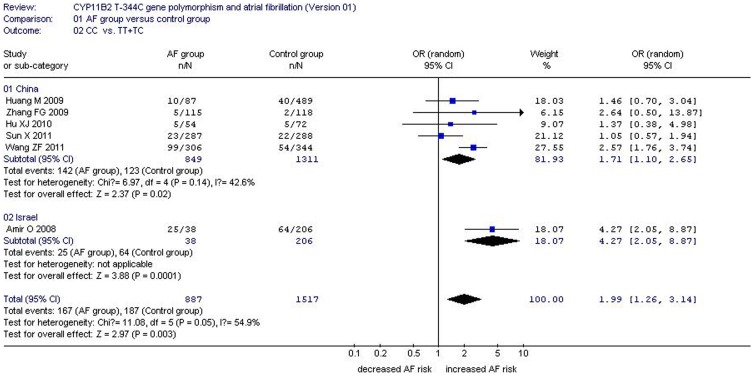
Forest plot of AF associated with CYP11B2 T-344C gene polymorphism stratified by study region under a recessive genetic model (CC vs. TT+TC).

**Table 3:** The meta-regression results among 6 studies under a recessive genetic model for the association of *CYP11B2* T-344C gene polymorphism and AF.

Item	Coefficient	Standard Error	T value	P value	95% Confidence Interval
Region	−2.669906	0.1079944	−24.72	0.026*	−4.042104∼−1.297707
TT1	0.0717151	0.0032119	22.33	0.028*	0.0309041∼0.1125261
TC1	−0.0597826	0.002674	−22.36	0.028*	−0.0937588∼−0.0258065
CC0	−0.0181076	0.0013512	−13.40	0.047*	−0.0352761∼−0.0009392
_cons	5.842676	0.215092	27.16	0.023*	3.109673∼8.575679

* :P<0.05.

Coefficient: regression coefficient.

The regression coefficients are the estimated increase in the lnOR per unit increase in the covariates. cons:constant item.

Region: study region; TT1: TT genotype number of AF group sample size; TC1: TC genotype number of AF group sample size; CC0: CC genotype number of control group sample size.

### Data Extraction

Data were drawn out according to a standard protocol. Repeated publications and studies violating the inclusion criteria or providing insufficient data were excluded. Same data from different studies were only adopted once. The extracted data comprised the following items: first author’s name, publication year, study region, number of genotypes, genotyping methods, study design, matching criteria, and total number of cases and controls.

**Table 4:** The meta-regression results among 6 studies under a dominant genetic model for the association of *CYP11B2* T-344C gene polymorphism and AF.

Item	Coefficient	Standard Error	T value	P value	95% Confidence Interval
Region	−0.5454399	0.0247974	−22.00	0.029*	−0.8605213∼−0.2303085
TT1	0.0111314	0.0011162	9.97	0.064*	−0.0030517∼0.0253144
TC1	−0.0077427	0.0009913	−7.81	0.081*	−0.0203378∼0.0048524
CC0	0.0009649	0.0001066	9.05	0.070*	−0.0003891∼0.0023188
_cons	0.5736956	0.0423936	13.53	0.047*	0.0350343∼1.112357

* :P<0.05.

Coefficient: regression coefficient.

The regression coefficients are the estimated increase in the lnOR per unit increase in the covariates. cons:constant item.

Region: study region; TT1: TT genotype number of AF group sample size; TC1: TC genotype number of AF group sample size; CC0: CC genotype number of control group sample size.

### Statistical Analyses

In the current meta-analysis, allelic (distribution of C allelic frequency of *CYP11B2* T-344C gene polymorphism), recessive (CC/TC+TT), dominant (TT/TC+CC), homozygous (CC/TT), heterozygous (TC/TT), and additive (C/T) genetic models were used. The relationship between *CYP11B2* T-344C gene polymorphism and AF was compared using odds ratios (ORs) and their corresponding 95% confidence intervals (CIs). Chi-square-based Q-test was used to calculate the between-study heterogeneity, with the significance level set at *P*<0.10 [13]. If heterogeneity existed among the individual studies, the pooled OR was estimated using the random-effects model (DerSimonian and Laird method) [14]. Otherwise, the fixed-effects model was adopted (the Mantel–Haenszel method) [15]. The pooled OR was determined using Z test, with the significance level set at *P*<0.05.

**Table 5:** The meta-regression results among 6 studies under a heterozygote genetic model for the association of *CYP11B2* T-344C gene polymorphism and AF.

Item	Coefficient	Standard Error	T value	P value	95% Confidence Interval
Region	0.712089	0.0088492	80.47	0.008*	0.5996493∼0.8245287
TC1	−0.0044425	0.0000753	−59.04	0.011*	−0.0053986∼−0.0034863
TT0	−0.0018085	0.0000383	−47.20	0.013*	−0.0022954∼−0.0013216
CC1	0.0035806	0.0001169	30.63	0.021*	0.0020953∼0.005066
_cons	−0.7071748	0.014178	−49.88	0.013*	−0.8873231∼−0.5270264

* :P<0.05.

Coefficient: regression coefficient.

The regression coefficients are the estimated increase in the lnOR per unit increase in the covariates. cons:constant item.

Region: study region; TT0: TT genotype number of control group sample size; TC1: TC genotype number of AF group sample size; CC0: CC genotype number of AF group sample size.

HWE was assessed using the Fisher’s exact test, with the significance level was set at *P*<0.05. The potential publication bias was estimated using the funnel plot. The funnel plot asymmetry was assessed by Egger’s linear regression test on the natural logarithmic scale of the OR, with the significance level set at *P*<0.05 [16]. Statistical analyses were performed by Revman 4.2 and STATA 11.0 software (StataCorp, College Station, TX, USA).

**Figure 4:**
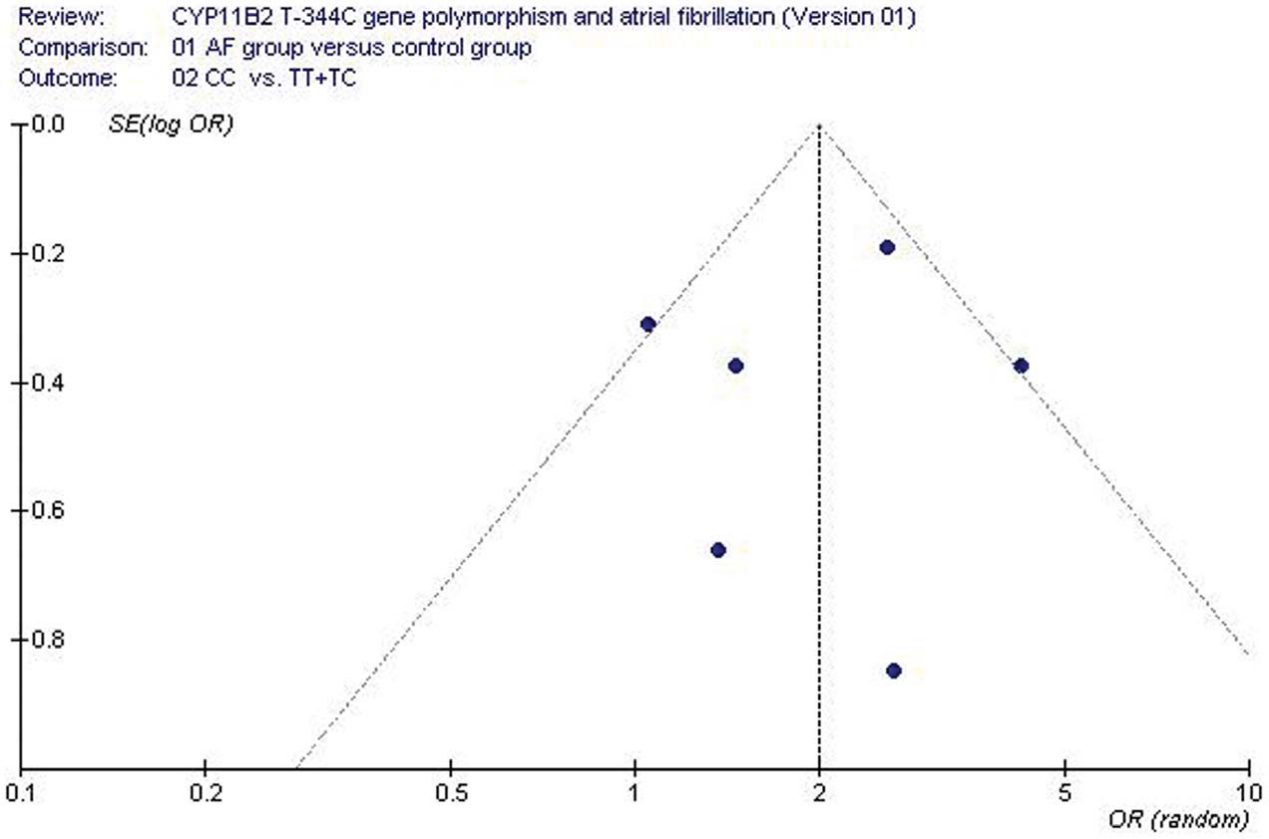
Funnel plot for studies of the association of AF associated with CYP11B2 T-344C gene polymorphism under a recessive genetic model (CC vs. TT+TC). The horizontal and vertical axis correspond to the OR and confidence limits. OR: odds ratio; SE: standard error.

## Results

### Studies and Populations

A total of 17 studies were retrieved, among which six met the inclusion criteria. Among the 11 excluded studies, five were character reviews and six were not associated with *CYP11B2* T-344C gene polymorphism or AF. No study was excluded for deviating from the HWE. The data were abstracted from 1,054 cases and 1,704 controls (Table 1, Supplement S2) [10]–[12], [17]–[19]. The included study regions were the Asian countries China and Israel.

### Pooled Analyses

Q-tests were used to test the heterogeneity among the studies. Given that the P value of Q-tests was more than 0.10 under the allelic (P_heterogeneity_ = 0.50), homozygous (P_heterogeneity_ = 0.813), and additive genetic models (P_heterogeneity_ = 0.416), the fixed-effects model was used. By contrast, the *P* value of Q-tests was less than 0.10 under the recessive (P_heterogeneity_ = 0.05), dominant (P_heterogeneity_ = 0.079), and heterozygous genetic models (P_heterogeneity_ = 0.001); thus, the random-effects model was adopted. The combined ORs and summary point of the association between *CYP11B2* T-344C and AF were calculated and obtained using Revman 4.2 and STATA 11.0 software. (Table 2, Figure 1–2).

A significant relationship between *CYP11B2* T-344C gene polymorphism and AF was found under the allelic (OR: 1.26, 95% CI: 1.11–1.42, P = 0.0002), recessive (OR: 1.99, 95% CI: 1.26–3.14, P = 0.003), dominant (OR: 0.903, 95% CI: 0.820–0.994, P = 0.036), homozygous (OR: 1.356, 95% CI: 1.130–1.628, P = 0.001), and additive (OR: 1.153, 95% CI: 1.070–1.243, P = 1.0×10^−10^) genetic models. However, no significant association between *CYP11B2* T-344C gene polymorphism and AF was found under the heterozygous genetic model (OR: 1.040, 95% CI: 0.956–1.131, P = 0.361) (Table 2, Figure 1–2).

Significant heterogeneity was found under the recessive (P = 0.05), dominant (P = 0.079), and heterozygous (P = 0.001) genetic models. The following meta-regression was subsequently conducted to explore the heterogeneity source. Under the recessive genetic model, the heterogeneity could be explained by study region (P = 0.026), TT (TT1, P = 0.028) and TC (TC1, P = 0.028) genotype numbers of AF group sample size, and CC genotype number of control group sample size (CC0, P = 0.047). According to study region, the whole population was separated into two subgroups. The studies performed in China belonged to subgroup 1 and the others into subgroup 2. In the subgroup analysis stratified by study region, a significant increase in AF risk was detected in both subgroups (subgroup 1: OR: 1.71, 95% CI: 1.10–2.65, P = 0.02, P_heterogeneity_ = 0.14; subgroup 2: OR: 4.27, 95% CI: 2.05–8.87, P = 0.0001). Heterogeneity disappeared in subgroup 1 (*I^2^* = 42.6%) (Tables 2 and 3, Figure 3).

Under the dominant genetic model, the heterogeneity could be explained by study region (P = 0.029), TT1 (P = 0.064), TC1 (P = 0.081), and CC0 (P = 0.070). According to study region, the whole population was divided into two subgroups under the recessive genetic model. In the subgroup analysis, a significant increase in AF risk was detected in subgroup 1 (OR: 0.897, 95% CI: 0.814–0.989, P = 0.029, P_heterogeneity_ = 0.049) but not in subgroup 2 (OR: 1.032, 95% CI: 0.601–1.770, P = 0.910) (Tables 2 and 4).

Under the heterozygous genetic model, the heterogeneity could be explained by study region (P = 0.008), TT0 (P = 0.013), TC1 (P = 0.011), and CC1 (P = 0.021). No significant increase in AF risk was observed in either of the subgroups (P>0.05) (Tables 2 and 5).

### Bias Diagnostics

The publication bias of the individual studies was evaluated by funnel plot and Egger’s test. No visual publication bias was found in the funnel plot (Figure 4). No significant difference was detected in the Egger’s test. This result indicates that no significant publication bias exists in the current meta-analysis (recessive genetic model, T = −1.41, P = 0.231).

## Discussion

In the current meta-analysis, a significant relationship was found between *CYP11B2* T-344C gene polymorphism and increased AF risk under the allelic (OR: 1.26), recessive (OR: 1.99), dominant (OR: 0.903), homozygous (OR: 1.356), and additive (OR: 1.153) genetic models. Under the heterozygous genetic model, the pooled OR was 1.040, suggesting no significant association between *CYP11B2* T-344C gene polymorphism and AF. Overall, a significant association exists between *CYP11B2* T-344C gene polymorphism and AF risk. Individuals with the C allele of *CYP11B2* T-344C gene polymorphism have higher risk for AF. The negative result under the heterozygous genetic model was possibly associated with the relatively smaller difference between cases and controls in TC genotype number than in CC genotype number proportion.

Considering the heterogeneity under the recessive, dominant, and heterozygous genetic models, a meta-regression was conducted to explore the heterogeneity source. In the heterogeneity source analysis, under the recessive genetic model, confounding factors such as study region (P = 0.026), TT1 (P = 0.028), TC1 (P = 0.028), and CC0 (P = 0.047) were shown to explain the possible heterogeneity source. The subgroup analysis stratified by study region demonstrated that the heterogeneity disappeared in the China subgroup (*P*>0.05). In addition, the association strength was reduced in the China subgroup (OR: 1.71, *P* = 0.02). Although the association found in the Israel subgroup was much stronger than that found in the China subgroup, the heterogeneity test was not applicable because only one study in the Israel subgroup was retrieved (OR: 4.27, *P* = 0.0001). Hence, study region was the main source of heterogeneity under the recessive genetic model.

Under the dominant genetic model, study region (*P* = 0.029), TT1 (*P* = 0.064), TC1 (*P* = 0.081), and CC0 (*P* = 0.070) could explain the heterogeneity source. Among these confounding factors, study region was the main source of heterogeneity. Based on the subgroup analysis stratified by study region, the China subgroup had more heterogeneity (*I^2^* = 58.1%, P_heterogeneity_ = 0.049). However, the association strength was enhanced (OR: 0.897, *P* = 0.029).

Under the heterozygous genetic model, study region (*P* = 0.008), TC1 (*P* = 0.011), TT0 (*P* = 0.013), and CC0 ( = 0.021) could explain the heterogeneity source. Among these confounding factors, study region was the key influencing factor. Although no difference between *CYP11B2* T-344C gene polymorphism and increased AF risk was found under this genetic model, the heterogeneity was more distinct in the China subgroup (*I^2^* = 79.5%, P_heterogeneity_ = 0.001) than in the whole population (*I^2^* = 76.1%, P_heterogeneity_ = 0.001).

AF is the most common and persistent arrhythmia in clinical practice. AF could increase the morbidity and mortality of heart failure and stroke. The occurrence of AF increases annually. It has dramatically elevated with increasing age, especially in chronic heart failure patients [20]. AF is known to be associated with atrial enlargement and fibrosis. Studies demonstrated that hyperaldosteronism is associated with hypertension, cardiovascular fibrosis, and electrolyte disturbances, including hypomagnesemia. In 2008, Sontia et al. found that aldosterone mediates blood pressure-independent renal and cardiovascular fibrosis and inflammation through Mg^2+^-sensitive pathways. Altered Mg^2+^ metabolism in hyperaldosteronism might be related to novel magnesium transporters and transient receptor potential melastatin cation channel downregulation; in addition, Mg^2+^ may have protective effects against the cardiovascular and renal damaging actions of aldosterone [21]. Appropriate Mg^2+^ supplement could help diminish the side effects of aldosterone and reduce the occurrence of AF.

CYB11B2 is the limiting-velocity enzyme of the last step in aldosterone biosynthesis. Therefore, the *CYB11B2* gene expression level is the key factor to regulate aldosterone secretion. The −344 polymorphism loci in the promoter region could play a regulatory role by combining with internal sterol growth factor-1 (SF-1). The -344C allele binds to SF-1 four times more than the T allele, which is associated with increased serum aldosterone level and AF occurrence [9]. This viewpoint was verified in the current meta-analysis.

The current meta-analysis faced some limitations. The studies included for the analysis were small (n = 6). Large-scale studies on the association between AF and *CYP11B2* T-344C gene polymorphism were still relatively inadequate. The CYP11B2 expression level was influenced not only by the *CYP11B2* T-344C gene polymorphism, but also by other genetic and environmental factors such as sympathetic nerve activation, inflammation state, and so on. Factors such as age, body mass index, and premeditation in the individual studies were not well matched yet.

In conclusion, *CYP11B2* T-344C gene polymorphism is significantly linked with increased AF risk. Patients with the C allele have higher risk for AF. The current conclusion may serve as a basis for the development of individual AF diagnosis and therapy strategies. Given the above limitations, more large-scale studies are needed to elucidate the significance of the verdict.

## Supporting Information

Supplement S1 **PRISMA 2009 Checklist.**
(DOC)

Supplement S2 **PRISMA 2009 Flow Diagram.**
(DOC)
