# Supplementary material for: CYP11B2 T-344C Gene Polymorphism and Atrial Fibrillation: A Meta-Analysis of 2,758 Subjects
Source: PLoS One. 2012 Nov 28;7(11):e50910. doi: 10.1371/journal.pone.0050910 (PMC3509071; doi:10.1371/journal.pone.0050910)
Supplement: Supplement S2 — PRISMA 2009 Flow Diagram. (DOC) [file pone.0050910.s002.doc]

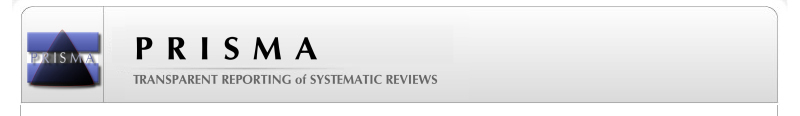
**PRISMA 2009 Flow Diagram**

**Screening**

**Included**

**Eligibility**

**Identification**

Records identified through database searching
(n =17 )

Additional records identified through other sources
(n =0 )

Records after duplicates removed
(n =17)

Records screened
(n =12 )

Records excluded for review characteristic
(n =5 )

Full-text articles assessed for eligibility
(n =12)

Full-text articles excluded for deviation from HWE (n =0 )

Studies included in qualitative synthesis
(n =6)

Records excluded for no association with CYP11B2 T-344C gene polymorphism or AF

(n =6)

Records excluded for repeated publication
(n = 0)
